# Correction: Fabrication of a reusable magnetic multi-walled carbon nanotube–TiO_2_ nanocomposite by electrostatic adsorption: enhanced photodegradation of malachite green

**DOI:** 10.1039/d2ra90112a

**Published:** 2022-11-14

**Authors:** Ghazale Daneshvar Tarigh, Farzaneh Shemirani, Nezam Seif Maz'hari

**Affiliations:** Department of Analytical Chemistry, University College of Science, University of Tehran P.O. Box 14155-6455 Tehran Iran ghdaneshvartarigh@yahoo.com Shemiran@khayam.ut.ac.ir +98 21 66495291 +98 21 66495291; Master of Science, University of Amirkabir Tehran Iran

## Abstract

Correction for ‘Fabrication of a reusable magnetic multi-walled carbon nanotube–TiO_2_ nanocomposite by electrostatic adsorption: enhanced photodegradation of malachite green’ by Ghazale Daneshvar Tarigh *et al.*, *RSC Adv.*, 2015, **5**, 35070–35079, https://doi.org/10.1039/C4RA15593A.

The authors regret that the authors affiliations were incorrectly shown in the original manuscript. The corrected list of affiliations is as shown above.

The Royal Society of Chemistry apologises for these errors and any consequent inconvenience to authors and readers.

## Supplementary Material

